# Computer Simulations of Cell Sorting Due to Differential Adhesion

**DOI:** 10.1371/journal.pone.0024999

**Published:** 2011-10-18

**Authors:** Ying Zhang, Gilberto L. Thomas, Maciej Swat, Abbas Shirinifard, James A. Glazier

**Affiliations:** 1 Cancer and Developmental Biology Laboratory, National Cancer Institute, Frederick, Maryland, United States of America; 2 Instituto de Física, Universidade Federal do Rio Grande do Sul, Porto Alegre, Brazil; 3 Biocomplexity Institute and Department of Physics, Indiana University, Bloomington, Indiana, United States of America; Université d'Evry val d'Essonne, France

## Abstract

The actions of cell adhesion molecules, in particular, cadherins during embryonic development and morphogenesis more generally, regulate many aspects of cellular interactions, regulation and signaling. Often, a gradient of cadherin expression levels drives collective and relative cell motions generating macroscopic cell sorting. Computer simulations of cell sorting have focused on the interactions of cells with only a few discrete adhesion levels between cells, ignoring biologically observed continuous variations in expression levels and possible nonlinearities in molecular binding. In this paper, we present three models relating the surface density of cadherins to the net intercellular adhesion and interfacial tension for both discrete and continuous levels of cadherin expression. We then use then the Glazier-Graner-Hogeweg (GGH) model to investigate how variations in the distribution of the number of cadherins per cell and in the choice of binding model affect cell sorting. We find that an aggregate with a continuous variation in the level of a single type of cadherin molecule sorts more slowly than one with two levels. The rate of sorting increases strongly with the interfacial tension, which depends both on the maximum difference in number of cadherins per cell and on the binding model. Our approach helps connect signaling at the molecular level to tissue-level morphogenesis.

## Introduction

The *cadherin* family of cell-adhesion membrane proteins plays a key role in both early and adult tissue morphogenesis [1]–[3]. Spatio-temporal variations in cadherin number and type help regulate many normal and pathological morphogenetic processes, including: neural-crest-cell migration [4], somite segmentation [5], [6], epithelial-to-mesenchymal transformations during tumor invasion and metastasis [7], [8], and wound healing [9], [10]. Many of these processes involve continuous variations in the expression level of a single type of adhesion molecule: During proximo-distal limb growth [11] and rostro-caudal body-axis elongation [12], adhesion gradients resulting from variations in the number of a single type of adhesion molecule may maintain cells' relative positions. *In vitro* and in experiments *in vivo*, when cells from different domains of a limb are mixed together, they can sort out according to their original positions [11], [13]. In *Drosophila*, an adhesion gradient drives the oocyte towards the posterior follicle cell, which expresses the highest level of DE-cadherin [14]. A cell-cell adhesion gradient along the dorso-ventral axis directs lateral cell migration during zebrafish gastrulation [15]. Thus, understanding the role of cadherins in creating and stabilizing tissue structures, especially the role of continuous variation in the level of a single cadherin, is crucial to understanding embryonic morphogenesis.

Steinberg's *Differential Adhesion Hypothesis* (*DAH*) originated the idea that cell sorting can result from variations in cell-cell adhesivity [16]–[19]. Cell sorting depends on the effective molecular binding strength between opposing cadherins, which in turn depends on their types and expression levels in each cell and potentially the cells' internal biochemistry and cytoskeletal structures [20]. Both differences in expression levels of a single type of cadherin [18], [19] and differences in the types of cadherins expressed [19], [21] can lead to sorting.

The relation between forces at the molecular level (pairs of cadherins), cell level (cell-cell adhesion), tissue level (surface tension) and cell sorting is more complicated than the simple physics suggested by the DAH. Experimental measurements of cadherin binding employing a variety of approaches have obtained widely differing estimates of the per-cadherin pair-binding force, cell-cell adhesion force and surface tension at the tissue level [19], [22]–[25]. In some experiments, the scaling between cadherin expression levels and surface tension, as given by equation (7), is quadratic (see equation (9)) [23]; in others, the scaling between cadherin expression levels and the cell-cell adhesion force is linear (see equation (10)) [19]. The cadherin organization within the cell membrane and the underlying cytoskeleton also change over a period of hours after two cells come into contact [3], [26]–[28]. Bindings between cadherin pairs differ for cadherins in different conformational states [3], *e.g.*, cadherin reorganization into adhesive patches on the cell membrane due to both passive diffusion and interaction with the actin cytoskeleton [26]–[28] can greatly increase the effective binding strength per cadherin pair between two cells. Cluster formation depends on the proper functioning of the actin cytoskeleton, so actin-disrupting drugs like cytochalasin-D and latrunculin greatly decrease cell-cell adhesivity [29].

Multiple transcriptional and post-translational signaling cascades can regulate cadherin expression levels, localization and per-cadherin binding strengths [3], [30]. In turn, cadherin binding can modify gene expression [3]. This complexity obscures the role of the cadherin-binding force in cell sorting [25]. As a result, different classes of experiments on specific types of cadherin have led to at least four simplified cadherin-binding models: the *linear-zipper model* (*LZM*) based on experiments on N-cadherin [31]–[34], the *cis-dimer model* (*CDM*) (equation (8)) based on experiments on E-cadherin [35], the *trans-homophilic-bond model* (*THBM*) (equation (9)) based on experiments on C-cadherin [36], and the *saturation model* (*SM*) (equation (10)), based on the observation that, for both the CDM and THBM models, when the cadherin binding between cells saturates, the number of bonds depends on the cell with the minimum cadherin concentration.

This paper therefore proposes a simple framework to explore how homotypic cadherin binding at the molecular level could produce intercellular adhesion and eventually determine cell sorting at the tissue level. We neglect complex spatial and temporal changes in cadherin behavior, assuming that cadherin distributions are uniform and constant on the cell membrane and that adhesion-strength per molecular bond is also time-independent (*i.e.*, we assume no conformational changes in molecular structure during a simulation). We then explore how the sorting configuration and rate depend on a few essential parameters in our models. Compared to the rate of sorting for an aggregate with two levels of a single cadherin, simulations with more intermediate levels sort more slowly but the sorting rate is similar for aggregates with the same number of cadherin levels for all binding models. The speed of sorting increases strongly with the interfacial tension, which depends both on the maximum difference in number of cadherins per cell and on the binding model.

## Methods

### Reaction-Kinetic Models of E-Cadherin Binding

The nature of cadherin-cadherin binding determines the way the cell-cell adhesion energy, depends on cells' cadherin surface densities, and thus the correct binding model to use in simulations of cell sorting. Since more recent mutagenesis studies do not support the linear-zipper model [3], we use the cis-dimer (*CDM*), the trans-homophilic-bond (*THBM*), and the saturation (*SM*) models to relate the cells' cadherin surface densities to the cell-cell adhesion energy.

The cis-dimer model (**CDM**) [35] assumes that cis-dimers first form on the surfaces of individual cells and that two dimers on apposing cells then bind together to form homophilic tetramers. Dimerization of monomers (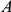 and 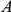 or 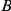 and 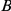) on individual cells' surfaces to form dimers 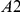 and 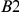 has the form:
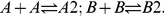
(1)Similarly, when the trans-tetramer 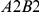 forms between dimers (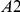 and 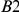) on two apposing cells, the reaction has the form:
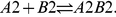
(2)


 We assume that the cadherin concentrations on the cells' surfaces are constant and that we can apply the Law of Mass Action. Dimerization and tetramerization quickly equilibrate if 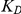 and 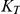, the *equilibrium dimerization* and *equilibrium tetramerization dissociation constants* are large and the cadherin concentrations, 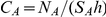 and 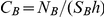, are lower than the dissociation constants [37]. Here 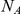 and 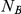 are the number of cadherin molecules distributed on the cell surfaces 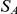 and 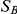, respectively, and 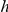 is the amplitude of cadherin fluctuations normal to the cells' surfaces. In this case, the total number of tetramers is less than the number of dimers, which in turn is less than the number of monomers. Then, the equilibrium concentration of tetramers in the CDM is, approximately,
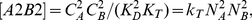
(3)where 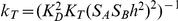 is the *tetramer effective equilibrium constant*.

According to the trans-homophilic-bond model (THBM) [36], cadherins bind individually between cells, so the concentration of bound pairs is given by:
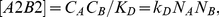
(4)where 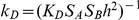 is the *dimer effective equilibrium constant*.

 Finally, for the saturation model (SM), which applies for strong clustering of cadherins, or large differences in the number of molecules per cell, the concentration of bound cadherin pairs is given by
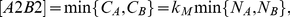
(5)where 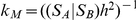 is the *effective equilibrium constant* and the surface 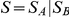 corresponds to the smaller of 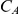 or 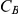.

We relate the concentration of cadherin pairs to the cell-cell *intercellular adhesion energy density* due to cadherin binding via the relation:
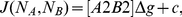
(6)where 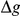 is the cadherin-cadherin-binding free-energy per cadherin bond [37], which is negative, since bond formation releases energy, and where 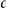 is the *energy density due to adhesion unrelated to cadherins* [19].

The *interfacial-tension density* over the contact area between two cells expressing different numbers of a single type of cadherin is defined [38], [39] as:
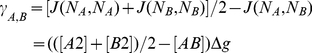
(7)For the three models just listed, equations (3–5), we have:
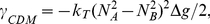
(8)

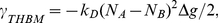
(9)

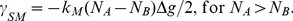
(10)


### Glazier-Graner-Hogeweg Simulations of Cell Sorting

To simulate cell sorting due to cell-cell adhesion, we used the *Glazier-Graner-Hogeweg* model (*GGH*) [40] (also known as the *Cellular Potts Model* [38], [39]). The GGH is a multi-cell, lattice-based model, which uses an *effective energy*, *H*, to describe the behavior of cells, for instance, due to cell-cell adhesion. GGH simulations agree quantitatively with simple cell-sorting and other experiments [41]–[49].

Cells in the GGH are extended domains of pixels (on a regular lattice, denoted 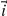), which share the same *cell index*, 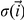. The effective energy governs how the lattice evolves as cells attempt to displace other cells by extending their pseudopodia [50]. At each step, we select a lattice site 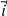 and change its index into the index of a neighboring lattice site 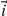 with probability:
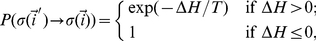
(11)where 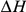 is the energy gain from the change and 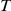 is the *intrinsic cell motility* corresponding to membrane fluctuations resulting from cytoskeleton fluctuations. If the lattice has 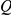 pixels, we define one *Monte Carlo Step* (*MCS*) to be 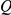 displacement attempts.

For a two-dimensional simulation of an aggregate containing cells expressing varying levels of a single type of cadherin, we assume that: (1) The effective energy between cells is due to cell-cell adhesion. (2) The cells have fixed and identical target volumes, membrane areas, and intrinsic motilities. (3) Cells do not grow, divide or die. (4) Cells are isotropic, so cadherins are uniformly distributed on the cell membrane and the cadherin concentration is constant in time. With these assumptions, the effective energy is:
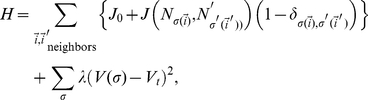
(12)where, 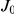 is the energy per unit contact area between two cells in the absence of cadherin binding, which may be positive since such cells may not cohere. 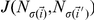 is the adhesion-energy per unit contact area between cells 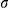 and 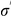 expressing 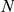 and 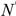 adhesion molecules, respectively. This term is always negative, since forming cadherin bonds decreases the effective energy. Sums go up to fourth nearest neighbors on a square lattice. 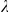, 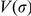, and 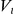 are the volume elasticity, actual volume and target volume of cell 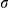, respectively. 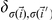 is the usual Kronecker delta function.

Each cell expresses a specific number of cadherins. The cell-cell adhesion energy relates to 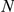 and 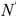 according to equation (6) together with equations (3), (4) or (5). Since we can rescale the energy by the intrinsic cell motility, we are free to pick the energy scale and set 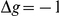.

The relative strengths of cell-cell adhesions result in net forces which act on each cell. Depending on the relative hierarchy of cell-cell adhesive interactions the generated forces can either drive or suppress cell sorting. Equation (13) is the condition for the sorting to occur.

**Figure 1:**
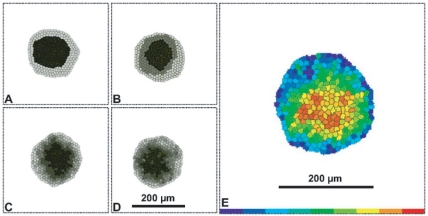
Typical simulated sorted configurations for aggregates of cells for the trans-homophilic-bond model (THBM). All images shown at time *t* = 999,000 MCS. In **A–D**, the gray-scale represents the cadherin-expression level. The darkest color (gray level = 0) represents the highest cadherin-expression level. The lightest color (gray level = 200) represents the lowest cadherin-expression level. The cell culture medium is white (gray level = 255). In (**E**), HSV colors represent the expression levels, (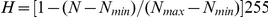, 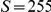, 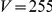), where 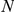 is the cadherin-expression level, and 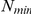 and 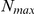 are the minimum and maximum cadherin-expression levels, respectively. **Red** (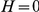) is the highest expression level, **blue** (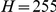) the lowest expression level. The cell culture medium is white. Sorting for: (**A**) 2 levels. (**B**) 3 levels. (**C**) 5 levels. (**D**) 9 levels. (**E**) Continuous levels. Cadherin expression ranges from 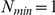 to 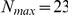. In all simulations, 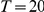 and 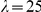.

Why does sorting occur for most of the conditions that we consider in this paper? For two cadherin levels with 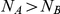, complete sorting requires that the less cohesive cell type wet the more cohesive cell type [39]:
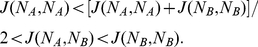
(13)Since 
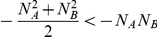
 for the THBM, 
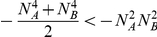
 for the CDM, and 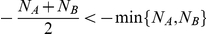 for the SM, the binding energies all satisfy the sorting condition. Therefore, cells should sort for all three binding models. Even cells with a continuous distribution of cadherin levels satisfy the sorting inequality, so cells with fewer adhesion molecules envelop cells with more adhesion molecules, which sort towards the center of the aggregate, creating an adhesion gradient, decreasing from the center to the periphery (Figure 1E), with a small amount of local mixing due to intrinsic cell motility. As mentioned above, sorting is a simple mechanism for cells to reach and maintain their positions during morphogenesis, *e.g.*, during limb outgrowth, in which cells maintain both their antero-posterior and proximo-distal positions through differential adhesion.

In an ideal, fully-sorted configuration, cells expressing the higher levels of cadherins will cluster together and round up into a solid sphere, surrounded by successive spherical shells of cells expressing successively lower levels of cadherins. To monitor the progress of cell sorting in our simulations, we define the heterotypic boundary length (*HBL*), the total contact length between cells with different cadherin levels, measured in pixels:
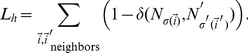
(14)The simulations time evolution gradually minimizes 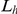.

If cells express multiple cadherin levels, 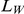, the heterotypic boundary length weighted by the energy differences between neighboring cells is a better metric for cell sorting. This weighted heterotypic boundary length (*WHBL*) is simply the total interfacial tension (equations (8–10)) multiplied by the lengths:
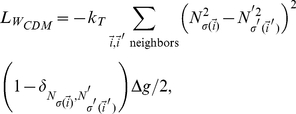
(15)

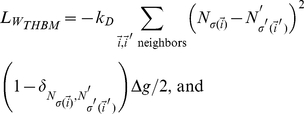
(16)

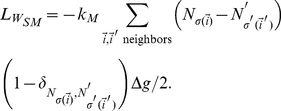
(17)


Different aggregates may have different maximum (initial) and minimum heterotypic boundary lengths (HBL) or weighted heterotypic boundary lengths (WHBL). To compare sorting in different aggregates, we normalize these lengths using the transformation:
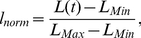
(18)where 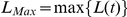, 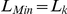 or 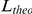, and 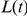 is the HBL or WHBL at time *t*. 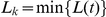 is the minimum value of HBL or WHBL over the typical simulation duration of 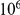 MCS. 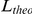 is the theoretical minimum HBL or WHBL for the fully sorted and rounded aggregate, assuming that the cells form perfect concentric rings with perimeters equal to 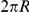 (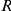 is the real radius of the ring of cells from the center of the aggregate). Experimentally, this value is easily calculated with digital imaging analysis, which gives us the total area of each type of cell. The *sorting relaxation time*, 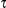, is the time at which the aggregate reaches its typical, maximally-sorted configuration. 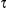 is defined via the relation:
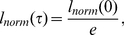
(19)


The *sorting rate*, 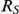, is the inverse of the sorting relaxation time:
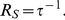
(20)


### Simulation Implementation

We first investigated sorting completeness for the trans-homophilic-bond model (THBM, equation (4)), with 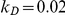, as we moved from two levels of cadherin expression towards a continuous distribution of levels (two, three, five, nine and continuous levels) with the same range of cadherin numbers, [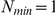, 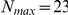]. The same range of cadherin expression numbers provides the same range of adhesion energies, independent of the number of levels.

We implemented our simulations using the open-source software package CompuCell3D (downloadable from http://www.compucell3d.org/) which allows rapid translation of biological models into simulations using a combination of CC3DML and Python scripting. We presented our simulation codes in Codes S1.

All our simulations for cell sorting use aggregates of 305 cells, close to the size of a 2D section of the 3D aggregates experimentally studied by Armstrong, Steinberg and others [18], [41], [51], which are about 200 microns in diameter. Each cell has a 25-pixel target volume, which sets the lattice length scale to approximately 2 microns per pixel. We begin with a circular-disk aggregate with cells randomly assigned cadherin expression numbers, with each allowed number having equal probability. Each simulation uses 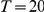 and runs for 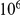 MCS, to allow for complete sorting for continuous variations of cadherin expression over the range [1, 23]. We set 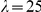, which allows patterns to evolve reasonably fast without large cell-volume or cell-surface-area fluctuations. Changing 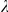 around this value does not greatly affect the relaxation of cells' shapes and positions. We further set 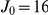 (in equation (12)) for all simulations. For different cadherin binding models and for the cadherin expression range [1, 23], we choose the values of 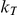, 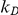 and 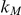 (according to equation (3–5)), so that cells neither pin to the lattice nor dissociate.

## Results

Figures 1A–E show final aggregates for cells expressing discrete or continuous levels of cadherins. Cells with higher expression (darker gray in Figures 1A–D, red in Figure 1E) assume more central positions, while cells with lower expression (lighter gray in Figures 1A–D, blue in Figure 1E) move to the periphery. For multiple discrete levels, cells follow a sorting hierarchy [17]; each layer of cells has a given expression number and surrounds the layer of cells with the next-higher level. For continuous levels, expression numbers decrease continuously from the center to the periphery of the aggregate (Figure 1E).

We investigated the evolution of the effective energy and the heterotypic boundary length (HBL)/weighted heterotypic boundary length (WHBL) for the THBM (equation (16)) in three cases:

1. Cells with different numbers of levels of cadherin expression, but the same range between maximum and minimum expression number.
2. Cells with different ranges between maximum and minimum expression number but with the same number of levels (two, for simplicity).
3. Cells with different motilities, but with the same cadherin levels.

We also investigated:

1. Cells with different cadherin binding models, but the same range between maximum and minimum expression number for two, five, nine and continuous levels.

**Figure 2:**
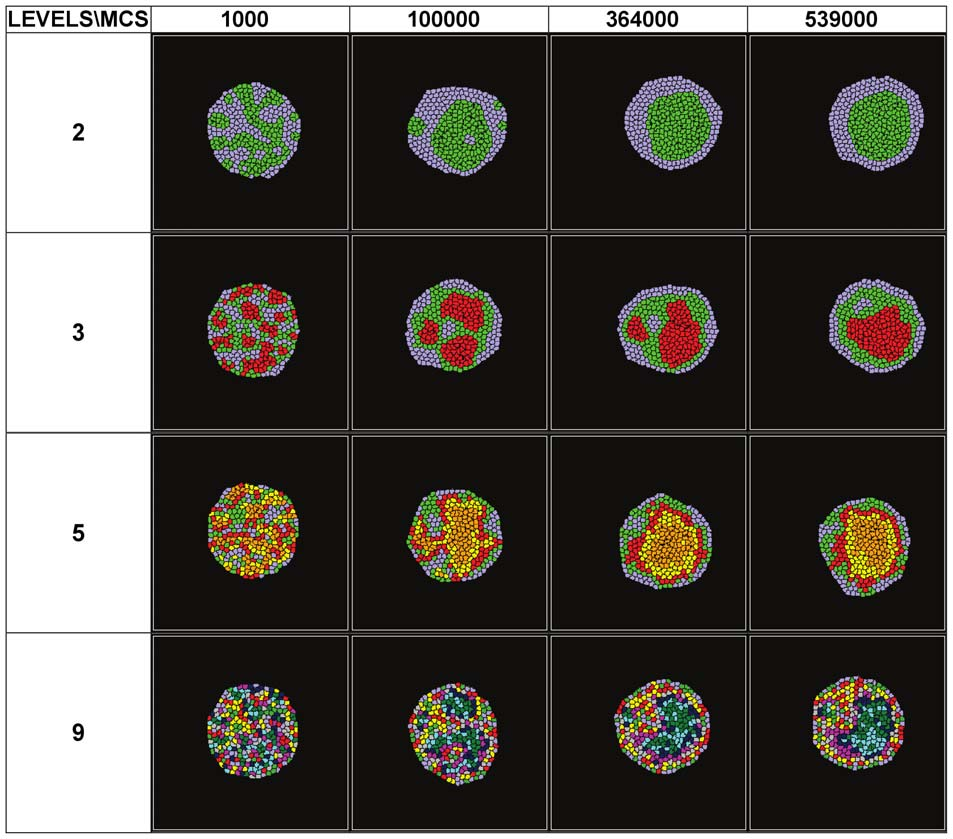
Simulation snapshots for aggregates with differing numbers of cadherin levels, with the same maximum to minimum expression range [1, 23], for the THBM. (See the animations in the supporting information.)

Figure 2 shows sets of snapshots of simulations for cell aggregates with the THBM (equation (4) with 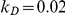, 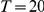, and 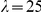) with cells expressing two [1, 23], three [1, 12, 23] , five [1, 6.5, 12, 17.5, 23], nine [1, 3.75, 6.5, 10.25, 12, 14.75, 17.5, 20.25, 23] cadherin levels. The corresponding animations are in: Movie S1, Movie S2, Movie S3, and Movie S4.

**Figure 3:**
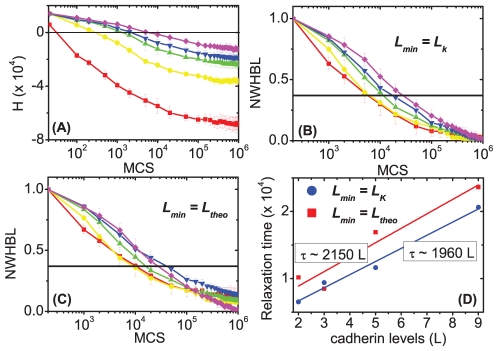
Evolution of the effective energies (H) and normalized weighted heterotypic boundary lengths (NWHBL) for aggregates with differing numbers of cadherin levels, with the same maximum to minimum expression range [1, 23], for the THBM. (**A**)**–**(**C**) 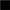 ▪– 2 levels; • – 3 levels; ▴ – 5 levels; ▾ – 9 levels; 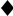 – continuous levels. The black horizontal lines mark 1/e. (**A**) Evolution of 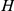. (**B**)**–**(**C**) Evolution of the NWHBL for the simulations in (**A**), with 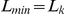 in (**B**) and 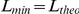 in (**C**). (**D**) Relaxation time *vs.* number of levels. ▪: 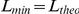. •: 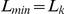. The graphs are calculated from ten simulation replicas.

Figure 3A shows the evolution of the effective energy 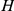 for the cell aggregates presented in Figure 2, and for cell aggregates with continuous cadherin levels in the range [1, 23] calculated using the THBM (equation (4) with 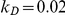, 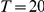, and 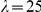). Figures 3B and 3C illustrate the evolution of the normalized weighted heterotypic boundary length (*NWHBL*) for the cell aggregates in Figure 3A, setting 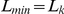 and 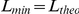, respectively. Aggregates with two or three levels sort quickly, while those with more levels take more time to sort (Figure 2D).

**Figure 4:**
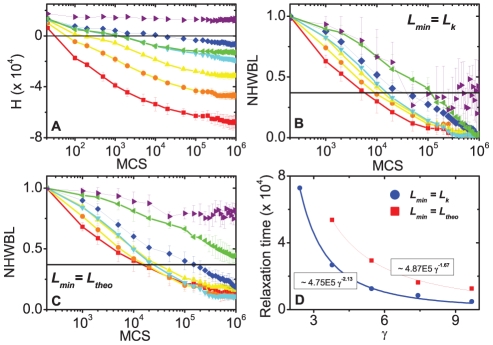
Evolution of the effective energies (H) and normalized weighted heterotypic boundary lengths (NWHBL) for aggregates expressing 2 cadherins levels modeled with the THBM. Expression ranges: ▪ – [1, 23]; • – [1, 20.25]; ▴ – [1, 17.50]; ▾ – [1,14.75]; ◂ – [12, 23]; 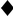 – [1, 12]; ▸ – [19.62, 23]. Black solid horizontal lines mark 1/e. (**A**) Evolution of 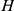. (**B**)**–**(**C**) Evolution of NWHBL for the simulations in (**A**) with: (**B**) 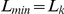, and (**C**) 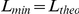. (**D**) Relaxation time *vs.* interfacial tension 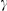. **Dots** – simulation, and **Lines** – fitting curves 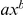. • – 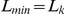; ▪ – 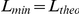. The error bars in the graphs are calculated from ten simulation replicas.

Figure 4A shows the evolution of the effective energy 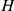 for aggregates with two cadherin levels, but different expression ranges: [1, 12], [1, 14.75], [1, 17.50], [1, 20.25], [1, 23], [12, 23], and [19.62, 23], also calculated using the THBM (equation (4) with 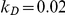, 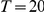, and 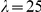). Figures 4B and 4C show the evolution of the NWHBL for the same aggregates, using 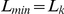 and 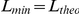, respectively. Sorting is quickest (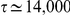 MCS) for aggregates with the widest cadherin expression range [1, 23], and is slowest (no complete sorting, 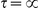) for aggregates with the smallest expression range [19.62, 23].

According to the theory of phase separation in liquids, the sorting rate for simple fluids is proportional to the interfacial tension divided by the viscosity' [52]. A similar relationship may hold for cell sorting [53]. Figure 4D plots the sorting relaxation time against the interfacial tension (equation 9) for the simulated aggregates in Figure 4A, and a power law (of form 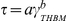, with 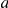 and 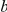 constants), fitting for both the cases 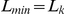 and 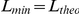, respectively:
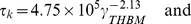
(21)

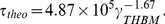
(22)The fitting is reasonable, since for 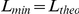, the adjusted coefficient of determination 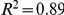, and for 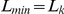, 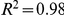, suggesting that the sorting relaxation time and interfacial tension may obey an approximate power law with an exponent 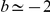.

**Figure 5:**
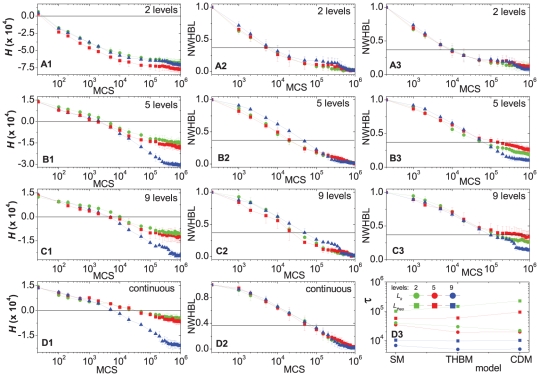
Evolution of the effective energies (H) and normalized weighted heterotypic boundary lengths (NWHBL) for aggregates with 2, 5, 9 or continuous cadherin levels using CDM, THBM or SM for the same expression range [1, 23]. In (**A1**)**–**(**A3**), (**B1**)**–**(**B3**), (**C1**)**–**(**C3**), and (**D1**)**–**(**D2**), **Red lines** ▪ – CDM; **Green lines** • – THBM; **Blue lines** ▴ – SM. In (**A2**), (**A3**), (**B2**), (**B3**) and (**C2**)**–**(**C4**), the time at which the heterotypic boundary length of a given simulation crosses the horizontal black line is defined as its relaxation time. In (**A2**), (**B2**), (**C2**) and (**D2**) 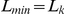. In (**A3**), (**B3**) and (**C3**) 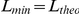. (**A1**), (**B1**), (**C1**) and (**D1**) Evolution of the 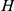 for aggregates with cells expressing 2, 5, 9 and continuous cadherin levels respectively. (**A2**), (**B2**), (**C2**) and (**D2**) Evolution of NWHBL for the aggregates in (**A1**), (**B1**), (**C1**) and (**D1**), respectively, with 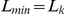. (**A3**), (**B3**) and (**C3**) Evolution of the NWHBL for the aggregates in (**A1**), (**B1**) and (**C1**) respectively, with 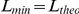. (**D3**) Relaxation time *vs.* bond model for different cadherin expression levels. **Blue** – 2 levels; **Red** – 5 levels; **Green** – 9 levels. **Circles** – 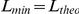. **Squares** – 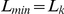. The error bars in the graphs are calculated from ten simulation replicas.

In Figure 5 we compare the evolution of the effective energy 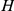 and of the NWHBL for the different cadherin binding models (CDM, THBM, and SM), with two, five, nine and continuous cadherin levels (the same levels as in Figure 3). We chose the effective equilibrium constants (see equations (8)–(10)), 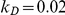, 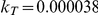, and 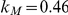, so the cell-cell adhesion energies fell in the same range, excluding changes in cell sorting rates due to differences in these ranges. Figure 5D3 shows that as the number of expression levels increases from 2 to 5 to 9, the relaxation time increases for each model.

For different models with the same cadherin expression levels, for two-level aggregates (Figure 5A1), sorting times are equal, as we expect because equations (8–10) give almost identical interfacial tensions. For aggregates with five and nine cadherin levels (Figures 5B1 and 5C1), sorting is more rapid for the saturation model (SM) and slowest for the trans-homophilic-bond model (THBM). The average minimum WHBLs are largest for the SM, but are the same for the cis-dimer model (CDM) and THBM. Since the weighted heterotypic boundary length (WHBL) is actually the interfacial tension, it is the main factor which determines the sorting rate.

**Figure 6:**
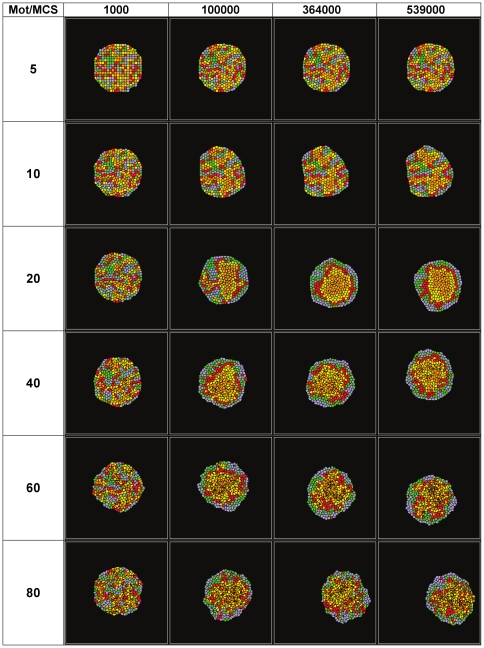
Simulation snapshots for aggregates with five levels [1, 6.5, 12, 17.5, 23] of cadherins and different cell motilities (5, 10, 20, 40, 60, 80), for the THBM. (See the animations in the supporting information.)

Figure 6 shows sets of snapshots of simulations for cell aggregates with the THBM (equation (4) with 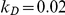, 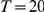, and 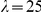) with five cadherin levels [1, 6.5, 12, 17.5, 23] and different cell motilities: 5, 10, 20, 40, 60, and 80. The corresponding animations are in: Movie S5, Movie S6, Movie S3, Movie S7, Movie S8, and Movie S9.

**Figure 7:**
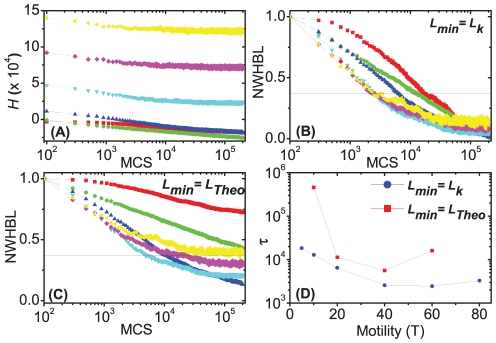
Evolution of the effective energies (H) and normalized weighted heterotypic boundary lengths (NWHBL) for aggregates with 5 cadherin levels and the same maximum to minimum expression range [1, 23] using the THBM with different motilities. ▪ – 5; • – 10; ▴ – 20; ▾ – 40; 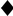 – 60; ◂ – 80. (**A**) Evolution of 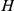. In (**B**) and (**C**) the time at which the heterotypic boundary length of a given simulation crosses the horizontal black line is defined as its relaxation time 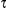. In (**B**) 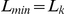 and in (**C**) 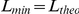. (**D**) Relaxation time vs. relative cell motilities. ▪ – 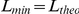; • – 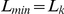. The error bars in the graphs are calculated from ten simulation replicas.

Figure 7 shows the effect of cell motility on the evolution of the effective energy and normalized WHBL for aggregates with two cadherin levels using the THBM (with 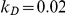 and 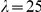). Figures 7A and 7B show the evolution of the effective energy for fixed 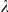. If the cell motility is very low (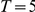), cells pin before reaching their lowest-energy positions and sorting is slow. As the motility grows, the aggregates sort faster (Figure 5A). However, if the cell motility is too large (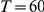 and 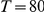), sorting is rapid but remains incomplete (Figures 7B, 7C and 7D).

When cells' expression of cadherin varies continuously, sorting still occurs, but more slowly than for discrete expression levels. The final configuration is imperfectly sorted since the intrinsic cell motility can overcome small differences in adhesion energy due to local missorting. The sorting rate depends on the interfacial tension rather than directly on the expression levels or the cadherin-binding model. Again, insufficient or excessive motility prevents complete sorting.

From the considerations above we can say that, although individually the sorting kinetics in aggregates with each binding model are sensitive to the number of cadherin levels and the energy range, all models have similar global behaviors. For each model, sorting is always faster for smaller numbers of cadherin levels, independent of the energy expression range. The dependence of sorting time and completeness on the number of cadherin levels is also similar for the three models, although the SM model seems to sort slightly faster and more completely for large numbers of cadherin levels. In the absence of experiments determining the model to use, the SM is computationally more efficient for larger aggregates.

Our results could be checked by experiments controlling cadherin expression. *E.g.* we could transfect a GFP-cadherin plasmid construct into normally non-adherent CHO cells, so the amount of cadherin in each cell would be proportional to its fluorescence intensity. For discrete levels we could use multiple fluorescent tags. Cotransformation with a nuclear-targeted fluorescent protein of a different color would allow real-time cell tracking to determine cell motilities and positions.

Using the interfacial boundary length as a measure of sorting is experimentally inconvenient because current automated image segmentation cannot accurately extract the interfacial lengths from a stack of images. Instead, measuring the autocorrelation of the intensity in experimental and simulation image stacks would be much simpler. To represent a nuclear-targeted label in our simulations we could place a dot at each cell's center of mass with an intensity proportional to the cell's the number of cadherins. To represent cytoplasmic labeling, we could fill the entire cell volume with an intensity corresponding to the cadherin level and similarly for membrane labeling, we could label the cell's contour.

**Figure 8:**
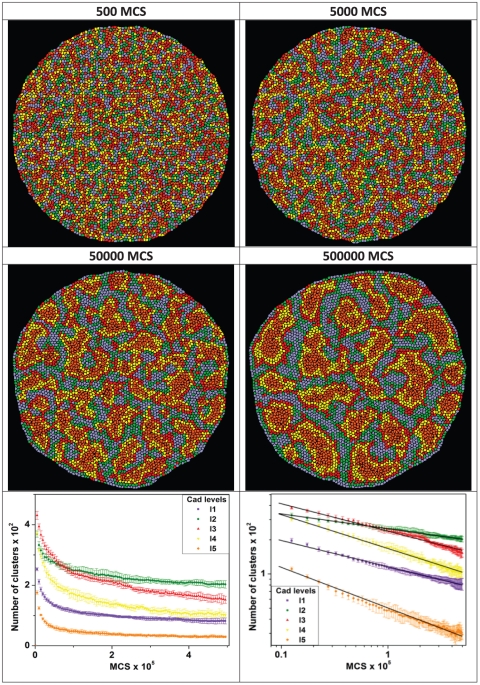
Clustering dynamics. First and second rows: snapshots taken from a 5000 cell aggregate simulation with five levels of cadherins [1(l1), 6.5(l2), 12(l3), 17.5(l4), 23(l5)] showing the dynamics of cluster formation. Bottom row: the left graph shows the evolution of the number of cluster for each cadherin level. The *log-log* graph (right) shows that the dynamics is adequately fitted by a power law of 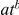, as indicated by the black lines. The error bars in the graphs are calculated from six simulation replicas.

An alternative measure of sorting would use a clustering algorithm to track the number and size of homotypic cell clusters. This approach is straightforward in CC3D and relatively easy to implement in experiments using K-Means or K-Median clustering algorithms, as described in [54]. Figure 8 shows an example of this procedure. We have used a bigger aggregate, with about 5000 cells in order to have a reasonable statistics. The cells have five levels of cadherins (as in Figure 6) and initially they are randomly distributed within the aggregate (top left snapshot). In our simple clustering algorithm, cells that express the same amount of cadherin and are in direct contact belong to the same cluster. The initial small clusters rapidly coalesce and form large clusters (top right and second row snapshots). The graphs at the bottom row show that the clustering rates decrease with time (left graph) and that they are adequately fitted by a power law of 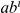, as can be seen from black lines in the *log-log* graph at right. Mean and error bars for these graphs are calculated from six simulation replicas.

Comparing any of these bulk cell-sorting measures for experiments and simulations would allow us to infer the specific binding mechanism in a particular experiment, information otherwise difficult to obtain.

## Discussion

At the beginning of a particular developmental phase, patterns of gene expression are often fuzzy initially, then gradually become distinct. Both changing cell identity and cell movement are possible mechanisms for refining initially fuzzy expression patterns or for fixing transient patterns of morphogens. Glazier *et al.* 2008 [49] and Watanabe *et al.* 2009 [55], found that, during somite segmentation, the fuzzy boundary formed by cells, disregarding positional cues and differentiating inappropriately, can reorganize to form a sharp boundary due to cell motility and differential adhesion. The sorting rate, and hence the rate of patterning, depend on the interfacial tensions, which in turn depend on the range of cadherin expression, equilibrium constants and free energies of cadherin bonds (see equations (8–10)). These mechanisms may act in parallel with, or coordinate with, other morphogenic mechanisms, such as Turing-type reaction-diffusion instabilities or Wolpertian threshold-based positional coding. Adhesion mechanisms act as an effective low-pass filter, reducing the effect of stochasticity in gene expression. During development, signaling cascades modulate cadherin expression. Because cell sorting is slow compared to fluctuations in gene-expression levels and because sorting rectifies noise into a stable gradient, transient fluctuations in cadherin expression will not change final morphology, increasing developmental robustness.

To provide better links/interplay between computer simulations and biological experiments, we would suggest carrying out measurements of the following key parameters [19], [22]–[25]: individual cell motilities, positions, contours and boundary lengths and tissue and single-cell level adhesion protein expression, elasticity and viscosity. While not always accessible, measurements of one or more adhesion-related parameters such as interfacial tension between cell aggregates, cell-cell adhesion forces or energies, molecular binding forces or energies and molecular binding and junction-formation kinetics would facilitate constructions of more realistic computer simulations. In particular, the ability to measure and then model temporal variation of adhesion related parameters is essential for simulations of complex developmental phenomena such as somitogenesis, limb growth, *etc…*. Therefore future measurements should concentrate on dynamics of intra and inter-cellular mechanisms (*e.g.* intercellular signaling and regulatory networks) related to cellular adhesion [3], [26]–[28].

Our studies based on the Glazier-Graner-Hogeweg model, investigated how homotypic cadherin binding at the molecular level affects cell-cell adhesion and determines cell sorting speeds at the tissue level. We have used three different microscopic models of cadherin-binding for discrete and continuous levels. The three binding mechanisms lead to similar cell-sorting behavior, although the saturation binding model is somewhat faster for larger aggregates with more cadherin levels. Sorting speed decreases with increasing numbers of cadherin levels. For classical sorting with two cadherin levels, sorting speed increases with the ratio between the two levels. Additionally, in each case a single optimum value for the cell motility results in the fastest sorting. Cell motilities above or below the optimum sort more slowly.

## Supporting Information

Codes S1 **XML configuration file and python scripts for the simulations presented in the text.**
(BZ2)

Movie S1 **Sorting of cells expressing two cadherin levels - [1, 23].**
(WMV)

Movie S2 **Sorting of cells expressing three cadherin levels - [1, 12, 23].**
(WMV)

Movie S3 **Sorting of cells expressing five cadherin levels - [1, 6.5, 12, 17.5, 23].**
(WMV)

Movie S4 **Sorting of cells expressing nine cadherin levels - [1, 3.75, 6.5, 10.25, 12, 14.75, 17.5, 20.25, 23]**.
(WMV)

Movie S5 **Sorting of cells expressing five cadherin levels (as above) and motility = 5.**
(WMV)

Movie S6 **Same five cadherin levels but motility = 10.**
(WMV)

Movie S7 **Same five cadherin levels but motility = 40.**
(WMV)

Movie S8 **Same five cadherin levels but motility = 60.**
(WMV)

Movie S9 **Same five cadherin levels but motility = 80.**
(WMV)
